# Mechanical Versus Biological Bentall Procedure: A Propensity-Score Matching Analysis of 548 Consecutive Patients

**DOI:** 10.3390/jcm14145105

**Published:** 2025-07-18

**Authors:** Antonella Galeone, Jacopo Gardellini, Fabiola Perrone, Venanzio Di Nicola, Giovanni Dian, Renato Di Gaetano, Giovanni Battista Luciani

**Affiliations:** 1 Department of Surgery, Dentistry, Pediatrics and Gynecology, Division of Cardiac Surgery, University of Verona, 37126 Verona, Italy; 2 School of Medicine, University of Verona, 37126 Verona, Italy; 3 Department of Cardiology, Azienda Sanitaria dell’Alto Adige, 39100 Bolzano, Italy

**Keywords:** Bentall procedure, mechanical prosthetic valve, biological prosthetic valve, propensity score matching

## Abstract

**Background/Objectives**: The Bentall procedure represents the gold standard therapy in patients with ascending aorta or aortic root aneurysm combined with aortic valve disease precluding a valve-sparing procedure. The aim of this study was to compare early and late outcomes in patients undergoing a Bentall procedure with either a biological or a mechanical valved conduit. **Methods**: All patients undergoing the Bentall procedure with either a biological or a mechanical valved conduit at our institution between 2001 and 2022 were retrospectively reviewed. A propensity-score (PS) matching analysis was performed to account for imbalances between the two groups. Clinical outcomes of interest included mortality and reintervention. **Results**: 548 patients underwent the Bentall procedure with a biological (n = 356, 65%) or a mechanical (n = 192, 35%) valved conduit during the study period. After PS-matching, two homogeneous groups of 154 patients were obtained, and no difference was observed in mean survival time between patients with mechanical Bentall and patients with biological Bentall (16 ± 0.8 vs. 16.3 ± 0.7 years, respectively; *p* = 0.72). Patients with a mechanical Bentall had a significantly higher mean survival time free from reintervention compared to patients with a biological Bentall (23.6 ± 0.4 vs. 21.4 ± 0.7 years, respectively, *p* = 0.02). PS-adjusted Cox regression showed that age >65 years, postoperative ECMO, and CVA were predictive risk factors of mortality. **Conclusions**: Bentall operation is a safe procedure for the treatment of ascending aorta and aortic root disease with good early and long-term survival and a low rate of reintervention. PS-matched analysis showed no difference in mortality between patients with a mechanical Bentall and patients with a biological Bentall; however, patients with a mechanical Bentall had a lower rate of reintervention.

## 1. Introduction

First described in 1968 by Bentall and De Bono [1] and then modified by Kouchoukos [2], the Bentall procedure allows a radical replacement of the aortic valve, the aortic root, and the ascending aorta with a valved conduit and thus represents the gold standard therapy in patients with ascending aorta or aortic root aneurysm combined with aortic valve disease precluding a valve-sparing procedure. The valved conduit may have a biological or mechanical prosthetic valve, and the choice between the two different types of prosthesis is currently based on the patient’s life expectancy, lifestyle and environmental factors, bleeding and thromboembolic risks related to anticoagulation, potential for surgical or transcatheter reintervention, and informed patient preference according to international guidelines [3]. The main concerns are related to the risk of thromboembolic and/or bleeding events and poorer quality of life due to lifelong anticoagulation for mechanical valves and the risk of structural valve dysfunction (SVD) requiring reintervention for biological valves. Previous studies of aortic valve replacement have had divergent results. A study involving 1001 matched pairs of patients 50 to 69 years of age who underwent isolated aortic valve replacement showed no significant difference in 15-year survival or stroke between biological and mechanical prosthesis [4]. Patients in the bioprosthetic valve group had a greater likelihood of reoperation but a lower likelihood of major bleeding [4]. Another study involving 287 matched pairs of patients aged 50 to 59 years showed that mechanical prostheses were associated with substantially lower mortality than biologic prostheses [5]. The risk of stroke was similar between the two groups; however, patients with bioprostheses had a higher risk of aortic valve reoperation and a lower risk of major bleeding [5]. Few studies compared the outcomes of biological versus mechanical valved conduits in patients undergoing the Bentall procedure [6,7,8,9,10,11] with controversial results; thus, the aim of this study was to compare early and late outcomes in patients undergoing a Bentall procedure with either a biological or a mechanical valved conduit using a propensity-score matching (PSM) analysis. The primary endpoint of this study was to evaluate the mortality in patients undergoing modified Bentall procedure; the secondary endpoints included cardiac reoperations for all causes.

## 2. Materials and Methods

This study was conducted in accordance with the Declaration of Helsinki and approved by the Ethics Committee of the Azienda Ospedaliera Universitaria Integrata of Verona (approval number: 13371; approval date: 13 March 2016).

All consecutive adult patients undergoing aortic valve, aortic root, and ascending aorta replacement with a mechanical or a biological valved conduit at our institution between January 2001 and December 2022 were included in this study. Patients’ preoperative characteristics, perioperative data, and in-hospital outcomes were extracted from patients’ paper-based and electronic medical records. Indications for surgery were based on the most recent available international recommendations [12].

All operations were performed through a median full sternotomy, using standard cardiopulmonary bypass (CPB) and cold blood or crystalloid cardioplegia. The replacement of the ascending aorta and aortic root was made according to the modified Bentall procedure or “Button technique” [13]. Briefly, the ascending aorta is transected, leaving a cuff of tissue distally for anastomosis to the aortic graft, is incised longitudinally just above the aortic valve commissures, and is transected at that level. Cold cardioplegia is infused directly into the coronary artery ostia through coronary cannulas. After the excision of the aortic valve leaflets, the three aortic sinuses are also excised, leaving a small rim. The coronary arteries are separated from the aortic sinus tissue with small buttons of full-thickness aortic wall and carefully mobilized to allow their attachment to openings in the aortic graft without tension [13]. When concomitant arch disease was present, moderate hypothermic circulatory arrest with anterograde cerebral perfusion was used for cerebral protection.

The choice between a biological or a mechanical valved conduit was made according to patient’s age, lifestyle, risk factors for bleeding or thrombosis, compliance with oral anticoagulants, and preference. All patients with mechanical valves were treated lifelong with warfarin at a target INR of 2.0 to 3.0. Patients with a biological valve were treated with warfarin for the first 3 months after surgery and then only with acetylsalicylic acid 100 mg once daily. In patients with a biological valve affected by atrial fibrillation, warfarin was not discontinued.

Follow-up data, including routine visits and subsequent hospitalization, were collected until May 2025 from cardiology reports and hospital records or via phone and e-mail contact with patients, family members, family physicians, and cardiologists. The follow-up time was calculated either to death or to the last verified contact with the patient. Clinical outcomes of interest included mortality and reintervention for infective endocarditis, aortic reasons, prosthetic valve dysfunction, or other causes. Mortality was defined as periprocedural (occurring ≤ 30 days after the index procedure or >30 days but during the index hospitalization), early (occurring > 30 days but ≤1 year after the index hospitalization), and late mortality (occurring > 1 year after the index hospitalization) according to Valve Academic Research Consortium 3 (VARC-3) [14]. Bioprosthesis valve dysfunction (BVD) was defined as the presence of SVD, non-SVD (NSVD), infective endocarditis, and thrombosis [14].

### Statistical Analysis

Categorical variables are expressed as numbers and percentages and compared with χ^2^ test. Continuous variables with a skewed distribution are presented as median and interquartile range and compared with Mann–Whitney U test.

The Kaplan–Meier method was used to draw survival curves; the log-rank test was used to compare survival among groups. The Reverse Kaplan–Meier survival curve was used to calculate follow-up rate. Completeness of the follow-up was measured according to Clark’s formula [15].

The PSM method was used to adjust for baseline differences between patients with biological Bentall and patients with mechanical Bentall and to reduce heterogeneity and confounding. The probability of being assigned to different surgical treatments was calculated from demographic and preoperative patients’ characteristics; the most clinically important variables were then entered into the PSM model.

Selected variables were age, sex, body mass index, body surface area, left ventricular ejection fraction, coronary artery disease, bicuspid aortic valve, infective endocarditis, acute aortic dissection, redo surgery, and emergency surgery. These covariates were used to compare both surgical techniques by logistic regression algorithm in 1-1 PSM. The nearest neighbor matching algorithm without replacement and a caliper size of 0.10 was used. Propensity matching models were assessed using balance diagnostics and standardized mean differences (SMD), with SMD < 0.1 reflecting a proper balance between groups.

Hazard ratios for mortality were determined by univariate and multivariate Cox proportional hazards regression analysis, with data presented as hazard ratios with 95% confidence interval (CI). A two-tailed *p* value < 0.05 was taken to indicate statistical significance. Statistical analysis was performed using SPSS version 26 (SPSS Inc., Chicago, IL, USA).

## 3. Results

### 3.1. Demography

**Figure 1:**
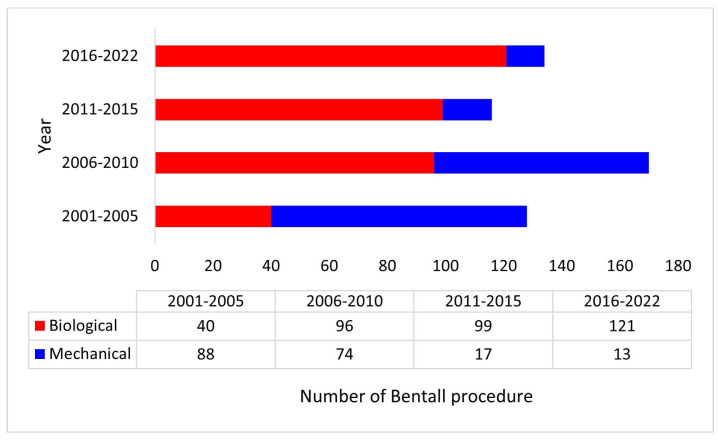
Distribution of the Bentall procedure with a biological or a mechanical valved conduit during the study period.

A total of 548 patients underwent a modified Bentall procedure with a biological (n = 356, 65%) or a mechanical (n = 192, 35%) valved conduit at our institution during the study period and were included in this study. Use of a biological valve conduit progressively increased during the study period (Figure 1).

Patients’ preoperative characteristics are illustrated in Table 1. Patients with a mechanical Bentall were significantly younger, had more acute aortic dissection, and underwent more frequent emergency operations than patients with a biological Bentall (Table 1). After PS-matching, two homogeneous groups of 154 patients were obtained with no difference in preoperative characteristics (Table 1). Of note, no difference was found between the two groups in perioperative complications such as low cardiac output syndrome (LCOS) requiring intra-aortic balloon pump (IABP) or extra-corporeal membrane oxygenation (ECMO), surgical re-exploration for bleeding, myocardial infarction (MI), sepsis, cerebrovascular accident (CVA), renal failure requiring continuous renal replacement therapy (CRRT), and grade III atrio-ventricular block requiring pacemaker (PM) implantation.

### 3.2. Subgroup Analysis

We performed a subgroup analysis with respect to the time of intervention. Patients were divided into two groups (group 1: 2001–2010 and group 2: 2011–2022) according to the year of intervention. Of note, patients in group 1 received significantly more mechanical valved conduits, had higher preoperative LVEF, and required less postoperative IABP and ECMO compared to patients in group 2 (Table 2).

A subgroup analysis was performed with respect to the patient’s age at the time of intervention. Of note, patients aged <65 years had significantly more bicuspid aortic valve and acute aortic dissection and less coronary artery disease compared to patients aged ≥ 65 years (Table 3). Patients aged <65 years also received more mechanical Bentall and underwent more emergent surgery and circulatory arrest. No difference was observed in perioperative complications between the two groups of patients.

### 3.3. Survival

Nine patients were lost during the follow-up period. The completeness of the follow-up was 98.4% according to Clark’s formula. The mean follow-up time was 14.4 ± 0.3 years. Overall, 250 patients (46%) died during the follow-up, 163 (46%) patients with a biological Bentall and 87 (45%) patients with a mechanical Bentall. We recorded 28 (5%) periprocedural deaths, 18 (3%) early deaths, and 204 (37%) late deaths (Table 4).

**Figure 2:**
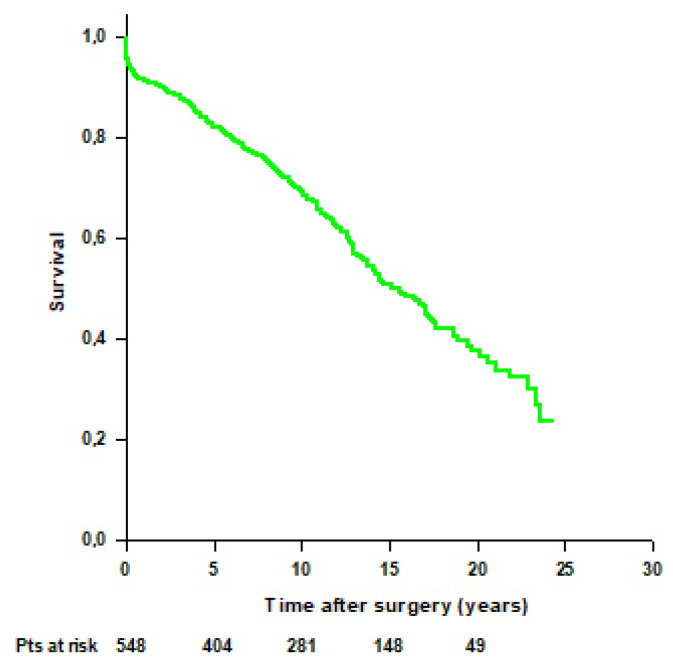
Patients’ survival after modified Bentall procedure in the whole cohort.

Mean survival time of the entire cohort was 14.8 ± 0.4 years, and survival rates were 95.8% at 30 days, 91.6% at 1 year, 82.2% at 5 years, 69.3% at 10 years, 50.7% at 15 years, and 37.6% at 20 years (Figure 2).

**Figure 3:**
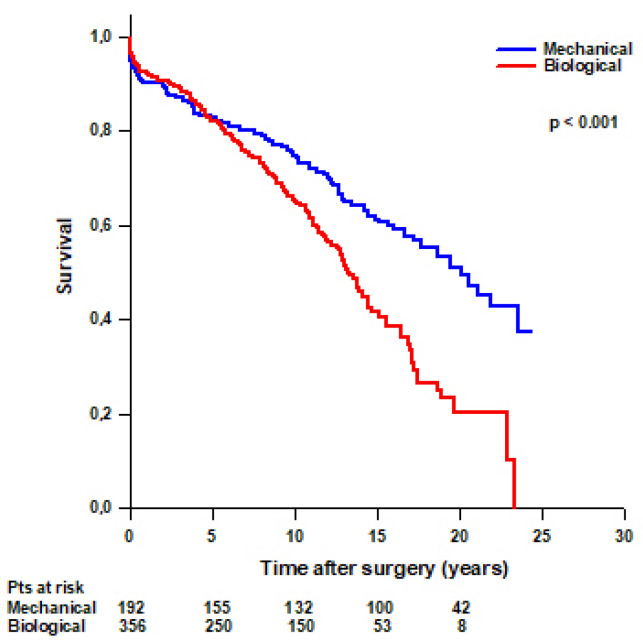
Patients’ survival after modified Bentall procedure with a mechanical or a biological valved conduit.

Mean survival time was significantly higher in patients with a mechanical Bentall compared to patients with a biological Bentall (16.5 ± 0.7 vs. 13 ± 0.5 years, respectively, *p* < 0.001) (Figure 3). Survival rates were 94.8% at 30 days, 90.1% at 1 year, 82.6% at 5 years, 74.8% at 10 years, 61.1% at 15 years, and 50.9% at 20 years in patients with a mechanical Bentall and 96.3% at 30 days, 92.4% at 1 year, 81.9% at 5 years, 65.4% at 10 years, 41.5% at 15 years, and 20.3% at 20 years in patients with a biological Bentall.

**Figure 4:**
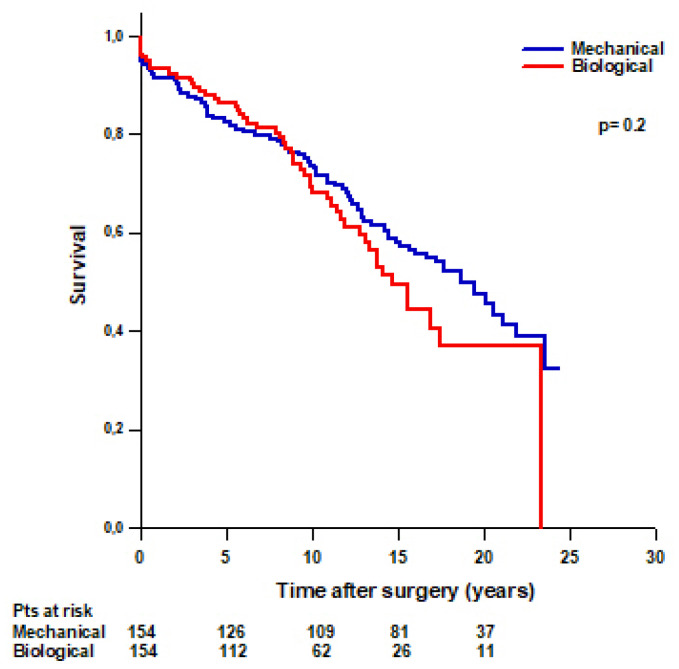
PS-matched patients’ survival after modified Bentall procedure with a mechanical or a biological valved conduit.

After PS-matching, we found that 55 (36%) patients with a biological Bentall and 78 (51%) patients with a mechanical Bentall died during the follow-up (Table 4). No difference was found in mean survival time between patients with a mechanical Bentall and patients with a biological Bentall (16 ± 0.7 vs. 14.8 ± 0.8 years, respectively, *p* = 0.2) (Figure 4). Survival rates were 94.8% at 30 days, 91.5% at 1 year, 82.4% at 5 years, 73.6% at 10 years, 58% at 15 years, and 47.3% at 20 years in patients with a mechanical Bentall and 96.1% at 30 days, 93.5% at 1 year, 86.5% at 5 years, 69.4% at 10 years, 49.3% at 15 years, and 37% at 20 years in patients with a biological Bentall.

**Figure 5:**
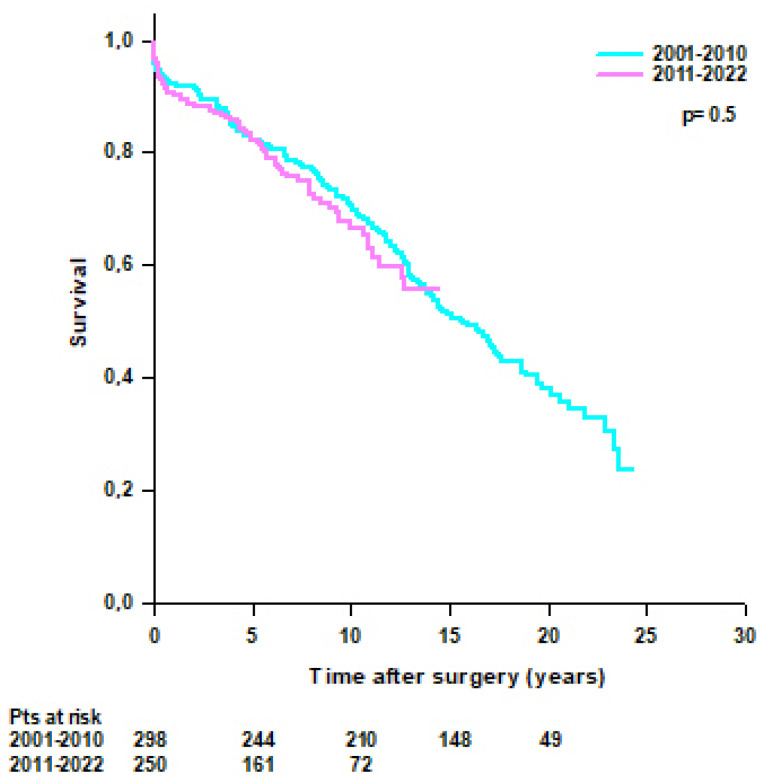
Patients’ survival after modified Bentall procedure according to the year of intervention (2001–2010 vs. 2011–2022).

No difference was found in survival rates between patients operated on from 2001 to 2010 and patients operated on from 2011 to 2022 (95.3% at 30 days, 92.3% at 1 year, 82.1% at 5 years, and 70.6% at 10 years vs. 96.4% at 30 days, 90.8% at 1 year, 82.9% at 5 years, and 66.7% at 10 years, respectively; *p* = 0.5) (Figure 5).

Univariate analysis was performed with several patients’ variables; significant variables on univariate analysis were entered in the Cox multivariate regression. Multivariate analysis showed that age >65 years, emergent surgery, postoperative LCOS requiring ECMO, and postoperative CVA were independent risk factors of mortality, while mechanical valved conduit had a protective effect (Table 5). PS-adjusted Cox regression confirmed that age >65 years, postoperative ECMO, and CVA were predictive risk factors of mortality (Table 5).

### 3.4. Reintervention

Twenty-four (4%) patients, sixteen (4%) with a biological Bentall and eight (4%) with a mechanical Bentall underwent reoperation during the follow-up for infective endocarditis (n = 12, 2%), aortic disease (n = 5, 1%), SVD (n = 3, 1%), and mitral regurgitation (MR) (n = 3, 1%) (Table 6).

**Figure 6:**
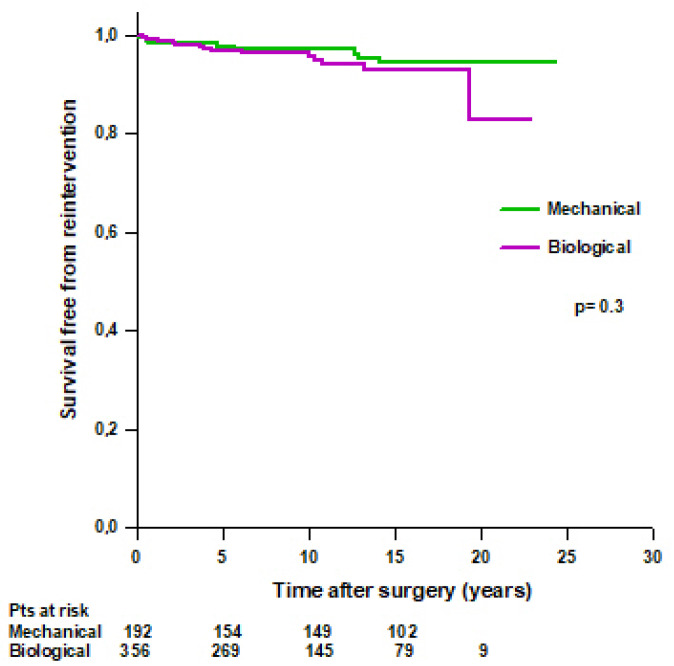
Patients’ survival free from reoperation after modified Bentall procedure with a mechanical or a biological valved conduit.

No difference was found in mean survival time free from reintervention between patients with a mechanical Bentall and patients with a biological Bentall (23.3 ± 0.4 vs. 21.4 ± 0.5 years, respectively, *p* = 0.3) (Figure 6). Survival rates free from reintervention were 97.7% at 5 years, 97% at 10 years, and 94.4% at 15 years in patients with a mechanical Bentall and 96.8% at 5 years, 95.7% at 10 years, 93% at 15 years, and 82.7% at 20 years in patients with a biological Bentall.

**Figure 7:**
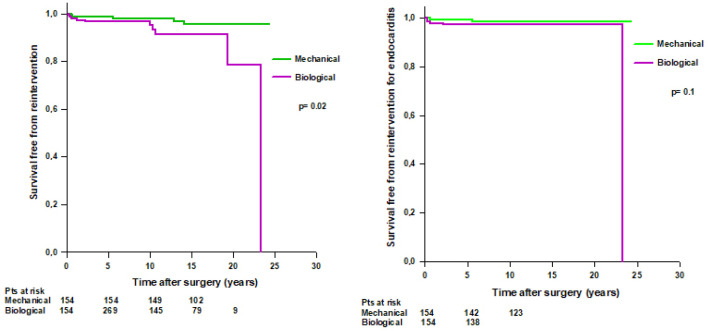
**Left**. PS-matched patients’ survival free from reoperation after modified Bentall procedure with a mechanical or a biological valved conduit. **Right**. PS-matched patients’ survival free from reoperation for infective endocarditis after modified Bentall procedure with a mechanical or a biological valved conduit.

After PS-matching, we found that 10 (6%) patients with a biological Bentall and 5 (3%) patients with a mechanical Bentall underwent reintervention during the follow-up (Table 6). Patients with a mechanical Bentall had a significantly higher mean survival time free from reintervention compared to patients with a biological Bentall (23.6 ± 0.4 vs. 21.4 ± 0.7 years, respectively, *p* = 0.02) (Figure 7 left). Survival rates free from reintervention were 98.6% at 5 years, 97.9% at 10 years, and 95.5% at 15 years in patients with mechanical Bentall and 96.5% at 5 years, 95% at 10 years, 91.4% at 15 years, and 78.3% at 20 years in patients with biological Bentall. No difference was found in survival free from reintervention for infective endocarditis between the two groups (Figure 7, right).

## 4. Discussion

Our report showed that the modified Bentall operation is a safe procedure with an overall mean survival time of 15 years and low rates of late reintervention. These excellent outcomes may justify the increase in the number of procedures performed over the last two decades observed in our study, despite the spread of valve-sparing procedures.

Current guidelines recommend mechanical prosthesis in patients aged <60 years and biological prosthesis in patients aged >65 years for a prosthesis in the aortic position [3]. Despite these recommendations, we observed an increase in the use of biological prosthetic valves over time, which is consistent with previously published reports highlighting a significant increase in the use of bioprosthetic valves over the last decades in all age groups [11,16]. Similarly, a recent observational study in more than 100,000 patients undergoing isolated primary aortic valve replacement (AVR) with a biological or a mechanical prosthesis also showed a decline in mechanical valve use from 20% to 10% over the 12-year period study [17]. However, in patients aged <60 years, receiving a mechanical AVR was independently associated with lower risk-adjusted all-cause mortality [17].

Accordingly, in our series, we found that long-term survival was significantly higher in patients with mechanical Bentall compared to patients with a biological Bentall in the unmatched population. However, the PS-adjusted survival showed no difference between the two groups, and propensity-adjusted Cox-regression analysis showed no relationship between the type of prosthesis and mortality at follow-up. Our results are in line with a previous study comparing biological versus mechanical Bentall procedures for aortic root replacement using a PS analysis of a series of 1112 patients [7]. Conversely, another study on more than 1200 patients showed that unadjusted and PS-adjusted ten-year mortality was higher in patients who received a mechanical Bentall [10]. However, these results may be due to the fact that in this series, although patients receiving a mechanical Bentall were younger, 33.3% were urgent or emergent procedures, and 19.2% were acute aortic dissections [10].

In our series, periprocedural mortality was 5% in both the unmatched and matched populations, which is consistent with previously reported in-hospital mortality ranging from 0.7% to 20% [4,5,6,7,8]. Similarly, a meta-analysis of 46 studies with 7629 patients reported an early mortality of 6% [18]. We did not find any important difference in the occurrence of early complications between the mechanical and biological Bentall, confirming the results of previously published reports [7,10]. Instead, other studies reported a higher incidence of postoperative ECMO and re-exploration for bleeding in patients with biological Bentall [9,11]. In our series, PS-adjusted Cox regression analysis showed that perioperative ECMO, postoperative CVA, and age >65 years were independent risk factors for mortality. Accordingly, a report on 597 patients showed age >65 years, coronary artery disease, and clots to be independent risk factors for late mortality after the Bentall procedure [6].

We found a low rate of late reintervention of 4% in the entire cohort, with patients with a mechanical Bentall having a significantly higher mean survival time free from reintervention compared to patients with a biological Bentall. The main cause of late reintervention in our series was infective endocarditis, which affected mostly bioprosthetic valves. These findings are consistent with a report based on a Swedish registry of more than 26,000 patients, finding out that the adjusted risk of both early and late endocarditis was higher in patients with bioprostheses compared to mechanical valves [19]. Likewise, a meta-analysis including 12 studies and almost 44,000 patients confirmed that bioprosthetic valves may be associated with a higher risk of infective endocarditis [20]. However, no difference was found in survival free from reintervention due to infective endocarditis in the PS-matched population between biological and mechanical Bentall, so we can speculate that the difference in survival free from reintervention observed between the two groups was mainly due to SVD, which affected only patients with a biological Bentall. Previous reports showed similar rates of reintervention but contrasting results on the cause of late reintervention after Bentall operation. Pantaleo et al. [7] reported 3% reintervention after Bentall operation, mainly due to prosthetic valve endocarditis affecting essentially bioprosthetic valves. However, the authors did not find any statistically significant difference in late reintervention between patients with a mechanical or a biological Bentall [7]. Perezgrovas-Olaria et al. [10] also reported an overall 4% late reintervention after the Bentall procedure, and 90% of late reinterventions were valve-related due to SVD. The authors observed significantly more reintervention in patients with a biological Bentall, mainly due to SVD [10]. In contrast, Lechiancole et al. did not observe any case of reoperation for SVD in their series despite the use of bioprosthesis, maybe for the short length of follow-up [8].

The avoidance of prosthetic valves and their complications in terms of thrombo-embolic, infective, and degenerative events has raised interest in valve-sparing procedures for aortic root disease; however, the superiority of this technique over the Bentall procedure is still debated. A recent meta-analysis showed that valve sparing surgery was associated with significantly greater survival at 15 years follow-up; however, it was also associated with a greater probability of reoperation within 5 years compared to Bentall-De Bono [21].

These results are in contrast with a previous meta-analysis, showing that long-term mortality was lower in valve-sparing procedures, while no difference was reported for late reoperation [22]. Another meta-analysis, including 34 studies published until June 2018, showed that the 5-year survival was greater in the valve-sparing procedure, and the 5-year reoperation rate was higher after Bentall-De Bono procedures [23]. A time-to-event reconstructed meta-analysis of six observational adjusted studies published until May 2023 revealed that long-term survival was higher in the valve-sparing procedure, and freedom from reoperation was greater in the valve-sparing procedure beyond 10 years [24].

Cryopreserved aortic homografts (CAHs) may be a valuable substitute for aortic root surgery, as they confer superior hemodynamic, avoidance of anticoagulation, and resistance to infections [25]. However, concerns exist about their durability and the risk of late reintervention for SVD; thus, the use of CAHs is usually reserved for native and prosthetic aortic valve endocarditis complicated by anular abscess and extensive destruction of the aortic root [26,27]. Non-frozen decellularized aortic homografts (DAHs) might present a valuable alternative for aortic root replacement due to their non-thrombogenicity, low immunogenicity, good haemodynamic characteristics, and low rate of adverse events [28]. A recent study aimed at comparing elective aortic root replacement with a mechanical valve or a DAH demonstrated significantly higher freedom from valve-related adverse events (including SVD, nSVD, bleeding, thrombosis, and embolization) at mid-term follow-up in patients treated with a DAH compared to patients receiving a mechanical Bentall [29].

## 5. Limitations

This study has limitations due to the retrospective observational design, where selection bias is unavoidable. In addition, this study covers a very long period, and pre- and postoperative management of the patients could have changed over time. Additionally, given the elderly population and the high overall mortality rates, the cumulative incidence of reintervention may be influenced by competing risks. Therefore, the study results should be interpreted with caution.

## 6. Conclusions

The modified Bentall operation is a safe procedure for the treatment of ascending aorta and aortic root disease with good early and long-term survival and a low rate of reintervention. This procedure is also characterized by a wide applicability in different settings of aortic pathologies, including aortic root aneurysm, acute aortic dissection, and aortic valve infective endocarditis. PS-matched analysis showed that the choice of either a mechanical or a biological valve graft has no influence on mortality, and the Bentall procedure provides a safe replacement of the aortic root in both elective and emergent surgery. However, patients with a mechanical Bentall had a lower rate of reintervention.

## Figures and Tables

**Table 1:** Pre-, intra-, and perioperative characteristics of the unmatched and PS-matched population.

	Unmatched Population			PS-Matched Population			
	Biological Bentall (n = 356)	Mechanical Bentall (n = 192)	*p*	Biological Bentall (n = 154)	Mechanical Bentall (n = 154)	*p*	SMD
**Preoperative characteristics**							
**Age, years**	68 (61–73)	59 (50–65)	<0.001	62 (55–68)	62 (53–66)	0.3	0.09
**Male sex**	292 (82%)	168 (88%)	0.1	131 (85%)	135 (88%)	0.6	0.1
**BMI**	26 (24–29)	27 (24–30)	0.1	26 (24–29)	27 (24–30)	0.4	0.06
**BSA, m^2^**	1.93 (1.8–2.06)	1.98 (1.83–2.09)	0.01	1.97 (1.83–2.1)	1.97 (1.84–2.08)	0.8	0.01
**LVEF (%)**	60 (55–60)	60 (52–60)	0.2	60 (55–60)	60 (55–60)	0.5	0.06
**Bicuspid aortic valve**	81 (23%)	57 (30%)	0.07	37 (24%)	43 (28%)	0.5	0.1
**Coronary artery disease**	46 (13%)	21 (11%)	0.5	25 (16%)	18 (12%)	0.3	0.1
**Acute aortic dissection**	43 (12%)	38 (20%)	0.02	26 (17%)	26 (17%)	0.9	0
**Infective endocarditis**	16 (4%)	8 (4%)	0.9	5 (3%)	5 (3%)	0.9	0
**Urgent/emergent surgery**	58 (16%)	45 (23%)	0.05	28 (18%)	32 (21%)	0.6	0.07
**Redo surgery**	43 (12%)	28 (15%)	0.4	24 (16%)	23 (15%)	0.9	0.02
**Intraoperative characteristics**							
**CPB time, min**	160 (135–203)	159 (135–207)	0.9	164 (141–224)	155 (134–201)	0.1	
**Aortic cross-clamping, min**	128 (109–149)	121 (106–151)	0.1	131 (110–153)	120 (106–144)	0.03	
**Circulatory arrest**	55 (15%)	44 (23%)	0.04	29 (19%)	35 (23%)	0.4	
**Aortic valve size, mm**	25 (25–27)	25 (25–27)	0.9	25 (25–27)	25 (25–27)	0.1	
**Aortic graft size, mm**	28 (28–30)	28 (26–30)	<0.001	28 (28–30)	28 (26–30)	<0.001	
**Arch replacement**	11 (3%)	5 (3%)	0.9	7 (5%)	4 (3%)	0.5	
**Emiarch replacement**	13 (4%)	5 (3%)	0.8	5 (3%)	5 (3%)	0.9	
**CABG**	54 (15%)	24 (13%)	0.4	31 (20%)	20 (13%)	0.1	
**MV repair/replacement**	10 (3%)	6 (3%)	0.9	5 (3%)	3 (2%)	0.7	
**Perioperative characteristics**							
**IABP/ECMO**	15 (4%)	6 (3%)	0.4	8 (5%)	3 (2%)	0.2	
**Revision for bleeding**	23 (6%)	17 (9%)	0.4	11 (7%)	15 (10%)	0.5	
**MI**	5 (1%)	2 (1%)	0.9	2 (1%)	2 (1%)	0.9	
**Sepsis**	7 (2%)	5 (3%)	0.8	6 (4%)	3 (2%)	0.4	
**CVA**	13 (4%)	8 (4%)	0.9	8 (5%)	8 (5%)	0.9	
**CRRT**	0	1 (1%)	-	0	1 (1%)	-	
**PM implantation**	1 (0.3%)	1 (1%)	0.8	0	1 (1%)	-	

**Table 2:** Pre-, intra-, peri-, and postoperative characteristics of the population according to the year of intervention.

	Group 1 2001–2010 (n = 298)	Group 2 2011–2022 (n = 250)	*p*
**Preoperative characteristics**			
**Age, years**	65 (57–71)	65 (55–71)	0.8
**Male sex**	256 (86%)	204 (82%)	0.2
**BMI**	27 (25–29)	26 (24–29)	0.3
**BSA, m^2^**	1.94 (1.8–2.03)	1.97 (1.84–2.1)	0.05
**LVEF (%)**	60 (55–60)	60 (51–60)	0.004
**Bicuspid aortic valve**	75 (25%)	63 (25%)	0.9
**Coronary artery disease**	40 (13%)	27 (11%)	0.6
**Acute aortic dissection**	41 (14%)	40 (16%)	0.5
**Infective endocarditis**	12 (4%)	12 (5%)	0.8
**Urgent/emergent surgery**	53 (18%)	50 (20%)	0.5
**Redo surgery**	38 (13%)	33 (13%)	0.9
**Intraoperative characteristics**			
**CPB time, min**	156 (135–195)	162 (136–221)	0.1
**Aortic cross-clamping, min**	123 (105–145)	129 (111–153)	0.04
**Circulatory arrest**	57 (19%)	42 (17%)	0.5
**Mechanical valved conduit**	162 (54%)	30 (12%)	<0.001
**Aortic valve size, mm**	25 (23–27)	25 (25–27)	0.007
**Aortic graft size, mm**	28 (26–28)	28 (28–30)	<0.001
**Arch replacement**	8 (3%)	8 (3%)	0.9
**Emiarch replacement**	7 (2%)	11 (5%)	0.2
**CABG**	42 (14%)	36 (14%)	0.9
**MV repair/replacement**	10 (3%)	6 (2%)	0.6
**Perioperative characteristics**			
**IABP/ECMO**	6 (2%)	15 (6%)	0.02
**Revision for bleeding**	19 (6%)	21 (8%)	0.4
**MI**	3 (1%)	4 (2%)	0.8
**Sepsis**	3 (1%)	9 (4%)	0.07
**CVA**	7 (2%)	14 (6%)	0.08
**CRRT**	0	1 (0.4%)	-
**PM implantation**	0	2 (1%)	-
**Postoperative characteristics**			
**Overall mortality**	178 (60%)	72 (29%)	<0.001
**Periprocedural mortality**	16 (5%)	12 (5%)	0.9
**Early mortality**	7 (2%)	11 (4%)	0.2
**Late mortality**	155 (52%)	49 (20%)	<0.001
**Reintervention**	10 (3%)	14 (6%)	0.2
**Infective endocarditis**	5 (2%)	7 (3%)	0.5
**Aortic disease**	1 (0.3%)	4 (2%)	0.2
**SVD**	2 (1%)	1 (0.4%)	0.8
**MR**	1 (0.3%)	2 (1%)	0.8

**Table 3:** Pre, intra, peri, and postoperative characteristics of the population according to the patient’s age.

	Patient’s Age <65 Years (n = 277)	Patient’s Age ≥65 Years (n = 271)	*p*
**Preoperative characteristics**			
**Age, years**	56 (49–61)	71 (68–75)	<0.001
**Male sex**	241 (87%)	219 (80%)	0.06
**BMI**	27 (24–30)	26 (24–29)	0.5
**BSA, m^2^**	1.74 (1.68–1.8)	1.71 (1.65–1.75)	<0.001
**LVEF (%)**	60 (55–60)	60 (52–60)	0.7
**Bicuspid aortic valve**	84 (30%)	54 (20%)	0.008
**Coronary artery disease**	22 (8%)	45 (17%)	0.003
**Acute aortic dissection**	54 (19%)	27 (10%)	0.003
**Infective endocarditis**	13 (5%)	11 (4%)	0.8
**Urgent/emergent surgery**	64 (23%)	39 (14%)	0.01
**Redo surgery**	36 (13%)	35 (13%)	0.9
**Intraoperative characteristics**			
**CPB time, min**	162 (134–222)	158 (136–191)	0.4
**Aortic cross-clamping, min**	124 (106–150)	127 (109–148)	0.3
**Circulatory arrest**	59 (51%)	40 (15%)	<0.001
**Mechanical valved conduit**	142 (51%)	50 (18%)	<0.001
**Aortic valve size, mm**	25 (25–27)	25 (23–27)	0.01
**Aortic graft size, mm**	28 (26–30)	28 (28–30)	0.9
**Arch replacement**	11 (4%)	5 (2%)	0.2
**Emiarch replacement**	8 (3%)	10 (4%)	0.7
**CABG**	29 (10%)	49 (18%)	0.01
**MV repair/replacement**	11 (4%)	5 (2%)	0.2
**Perioperative characteristics**			
**IABP/ECMO**	12 (4%)	9 (3%)	0.6
**Revision for bleeding**	26 (9%)	14 (5%)	0.08
**MI**	2 (1%)	5 (2%)	0.4
**Sepsis**	7 (3%)	5 (2%)	0.8
**CVA**	8 (3%)	13 (5%)	0.3
**CRRT**	1 (0.4%)	0	-
**PM implantation**	1 (0.4%)	1 (0.4%)	0.9
**Postoperative characteristics**			
**Overall mortality**	91 (33%)	159 (59%)	<0.001
**Periprocedural mortality**	16 (6%)	12 (4%)	0.5
**Early mortality**	9 (3%)	9 (3%)	0.9
**Late mortality**	66 (24%)	138 (51%)	<0.001
**Reintervention**	18 (6%)	6 (2%)	0.02
**Infective endocarditis**	8 (3%)	4 (1%)	0.4
**Aortic disease**	4 (1%)	1 (0.4%)	0.3
**SVD**	3 (1%)	0	-
**MR**	3 (1%)	1 (0.4%)	0.6

**Table 4:** Overall, periprocedural, early, and late mortality of the unmatched and PS-matched population.

	Unmatched Population			PS-Matched Population		
	Biological Bentall (n = 356)	Mechanical Bentall (n = 192)	*p*	Biological Bentall (n = 154)	Mechanical Bentall (n = 154)	*p*
**Overall mortality**	163 (46%)	87 (45%)	0.9	55 (36%)	78 (51%)	0.01
**Periprocedural mortality**	15 (4%)	13 (7%)	0.2	6 (4%)	9 (6%)	0.5
**Early mortality**	12 (3%)	6 (3%)	0.9	4 (3%)	4 (3%)	0.9
**Late mortality**	136 (38%)	68 (35%)	0.5	45 (29%)	65 (42%)	0.02

**Table 5:** Unadjusted and PS-adjusted Cox regression univariate and multivariate analysis.

	Unmatched Population				PS-matched Population			
	Univariate Analysis		Multivariate Analysis		Univariate Analysis		Multivariate Analysis	
Variable	Hazard Ratio (95% CI)	*p*	Hazard Ratio (95% CI)	*p*	Hazard Ratio (95% CI)	*p*	Hazard Ratio (95% CI)	*p*
**Age >65 years**	2.48 (1.89–3.24)	<0.001	2.22 (1.66–2.96)	<0.001	1.92 (1.36–2.7)	<0.001	1.74 (1.22–2.47)	0.002
**Sex, female**	1.16 (0.84–1.61)	0.35			1.01 (0.62–1.64)	0.9		
**BAV**	0.62 (0.46–0.84)	0.003	0.76 (0.56–1.04)	0.09	0.55 (0.36–0.84)	0.006	0.63 (0.41–0.98)	0.04
**Coronary artery disease**	1.36 (0.96–1.93)	0.08			1.1 (0.68–1.8)	0.68		
**Aortic dissection**	1.19 (0.84–1.68)	0.31		-	1.22 (0.78–1.9)	0.36		
**Infective endocarditis**	1.84 (1.10–3.05)	0.01	1.36 (0.76–2.42)	0.28	1.75 (0.77–3.98)	0.18		
**Redo surgery**	1.24 (0.87–1.77)	0.22			1.58 (1.01–2.47)	0.04	1.39 (0.88–2.18)	0.14
**Urgent/emergent surgery**	1.44 (1.06–1.94)	0.01	1.43 (1.09–2)	0.03	1.38 (0.91–2.08)	0.12		
**Mechanical valved conduit**	0.55 (0.41–0.72)	<0.001	0.64 (0.48–0.86)	0.003	0.81 (0.56–1.16)	0.26		
**IABP**	2.49 (1.17–5.3)	0.01	1.45 (0.59–3.55)	0.4	1.96 (0.62–6.17)	0.24		
**ECMO**	6.58 (3.08–14.02)	<0.001	3.72 (1.56–8.86)	0.003	5.68 (2.08–15.47)	<0.001	5.03 (1.79–14.15)	0.002
**Postoperative CVA**	3.79 (2.34–6.16)	<0.001	3.37 (2.02–5.62)	<0.001	3.58 (2–6.4)	<0.001	2.9 (1.59–5.26)	<0.001
**Reintervention**	0.79 (0.42–1.49)	0.47			0.85 (0.37–1.94)	0.7		

**Table 6:** Reintervention of the unmatched and matched populations.

	Unmatched Population			PS-Matched Population		
Characteristics	Biological Bentall (n = 356)	Mechanical Bentall (n = 192)	*p*	Biological Bentall (n = 154)	Mechanical Bentall (n = 154)	*p*
**Reintervention**	16 (4%)	8 (4%)	0.9	10 (6%)	5 (3%)	0.3
**Infective endocarditis**	9 (3%)	3 (2%)	0.6	5 (3%)	2 (1%)	0.4
**Aortic disease**	3 (1%)	2 (1%)	0.9	2 (1%)	0	-
**SVD**	3 (1%)	0	-	3 (2%)	0	-
**MR**	1 (0.3%)	3 (2%)	0.2	0	3 (2%)	-

## Data Availability

The data presented in this study are available upon request from the corresponding author.

## Abbreviations

The following abbreviations are used in this manuscript:

AVR	Aortic valve replacement
BMI	Body mass index
BSA	Body surface area
BAV	Bicuspid aortic valve
CABG	Coronary artery bypass-grafting
CAH	Cryopreserved aortic homograft
CAV	Cerebrovascular accident
CI	Confidence Interval
CPB	Cardiopulmonary bypass
CRRT	Continuous renal replacement therapy
DAH	Decellularized aortic homograft
ECMO	Extra-corporeal membrane oxygenation
IABP	Intra-aortic ballon pump
INR	International normalized ratio
LCOS	Low cardiac output syndrome
LVEF	Left ventricular ejection fraction
MI	Myocardial infarction
MR	Mitral regurgitation
MV	Mitral valve
PM	Pacemaker
PSM	Propensity score matching
SMD	Standardized mean difference
SVD	Structural valve degeneration
